# The Toxins of *Beauveria bassiana* and the Strategies to Improve Their Virulence to Insects

**DOI:** 10.3389/fmicb.2021.705343

**Published:** 2021-08-26

**Authors:** Haiyang Wang, Hui Peng, Wenjuan Li, Peng Cheng, Maoqing Gong

**Affiliations:** ^1^Shandong Institute of Parasitic Diseases, Shandong First Medical University & Shandong Academy of Medical Sciences, Jining, China; ^2^College of Forensic Medicine and Laboratory Medicine, Jining Medical University, Jining, China

**Keywords:** *Beauveria bassiana*, virulence, toxins, biological insecticides, insect

## Abstract

The long-term and excessive usage of pesticides is an enormous burden on the environment, which also increases pest resistance. To overcome this problem, research and application of entomopathogenic fungi, which are both environmentally friendly and cause lower resistance, have gained great momentum. Entomopathogenic fungi have a wide range of prospects. Apart from *Bacillus thuringiensis*, *Beauveria bassiana* is the most studied biopesticide. After invading insect hosts, *B. bassiana* produces a variety of toxins, which are secondary metabolites such as beauvericin, bassianin, bassianolide, beauverolides, tenellin, oosporein, and oxalic acid. These toxins help *B. bassiana* to parasitize and kill the hosts. This review unequivocally considers beauveria toxins highly promising and summarizes their attack mechanism(s) on the host insect immune system. Genetic engineering strategies to improve toxin principles, genes, or virulent molecules of *B. bassiana* have also been discussed. Lastly, we discuss the future perspective of *Beauveria* toxin research, including newly discovered toxins.

## Introduction

Although chemical insecticides have been remarkably effective against agricultural pests and medically important arthropods, these often have problems of insecticide resistance and environmental damage (Naqqash et al., 2016). Therefore, bioinsecticides, such as those produced from entomopathogenic fungi, are rapidly emerging as prime substitutes (Zhang et al., 2020b). Notably, filamentous fungi, a major branch of eukaryotes, emerged during a long evolutionary period. Studies and phylogenetic data over the last four decades indicate that convergent evolution enhanced fungal virulence to most pests and medically important arthropods. This is the prominent feature of several fungal lineages, which has drawn wide attention (Vega, 2008; Zheng et al., 2013; Wang et al., 2021). Apart from the extracted active ingredients from filamentous fungi, biopesticides also refer to other pesticides that are derived from natural sources such as animals, plants, bacteria, and certain minerals (Mathur, 2013). As of April 2016, there are 299 biopesticide active ingredients and 1,401 active biopesticide products registered by the United States Environmental Protection Agency (Kupfer and Mcmanus, 2008). Among these, *Metarhizium anisopliae*, *Beauveria bassiana*, and *Bacillus thuringiensis* are the most effective biological control agents against mosquito vectors (Montalva et al., 2016). Apart from *B. thuringiensis*, *B. bassiana* is the most commonly used biopesticide that can be effectively transmitted (Baldiviezo et al., 2020).

*Beauveria bassiana*, first isolated from silkworm cadavers by Agostino Bassi in the 19th century, can invade more than 200 species of insects in six orders and 15 families (Nakahara et al., 2009). It multiplies rigorously, producing a variety of toxins causing exogenous infections (Chelico and Khachatourians, 2008; Naqqash et al., 2016).

Over the past decade, the emergence of genomics, proteomics, and immense advances in molecular biology and genetic techniques has helped in the identification of several proteins or regulatory factors related to stress responses and/or fungal virulence. The studies in insect host(s), which is a more suitable research model, could also capture even the transient interaction with fungi toxins. In general, the host–fungus biological interactions are more prominent in the host insect and can be further magnified for research purposes (Joop and Vilcinskas, 2016).

In recent years, several research papers, including reviews, about the insecticidal effects of entomopathogenic fungi have been published; most of these discuss *B. bassiana* (Khan et al., 2016; Wang et al., 2016; Chu et al., 2017). Therefore, to avoid repetition and bring out a new perspective, herein we unequivocally focus on the *Beauveria* toxin. We summarize how it attacks the insect host immune system and discuss genetic engineering strategies to improve its toxicity, with a special focus on underlying genetic and molecular mechanisms of fungal virulence. Furthermore, we mention the newly discovered toxins with novel insecticidal activity. We believe that this review would help novice researchers to understand the latest findings in the field to speed up their research endeavors.

## The Process of Fungal Pathogenesis and Toxicity

**FIGURE 1:**
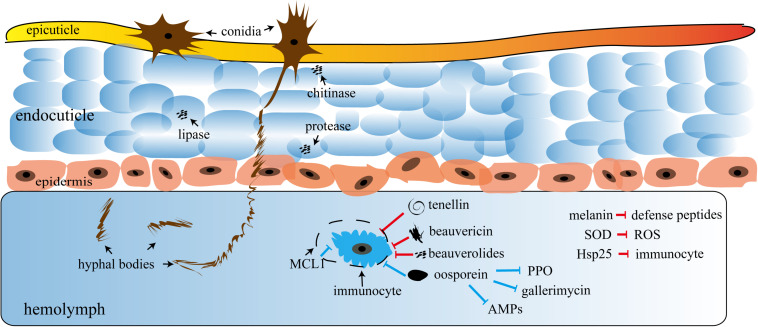
A schematic of fungal invasion. AMPs, antimicrobial peptides; PPO, polyphenol oxidase; MCL1, myeloid cell leukemia sequence, an antiapoptotic protein; SOD, superoxide dismutase; ROS, reactive oxygen species; Hsp25, heat shock factor 25. The conidia firstly penetrate the epicuticle to reach the endocuticle of the insect and then release plenty of hydrolases, including chitinase, protease, and lipase, to rapidly disintegrate the cuticle for more conidia intrusion. Hyphal bodies that pass through the epidermis intrude into the hemolymph of the insects and secrete plenty of insecticidal substances, such as oosporein, beauvericin, beauverolides, and tenellin, most of which either suppress the immunocyte or directly destroy the hemolymph. The substances mentioned in the bottom right of the figure suppress the immune system. The red and blue arrows represent the direct and indirect inhibition responses, respectively.

Insect cuticle penetration is the first step of entomopathogenic fungi infection, which involves mechanical forces, cuticle-degrading enzymes (chitinase, lipase, protease, etc.), and hyphae-produced specific infection structures (appressoria) that penetrate the host cell and proliferate (Chelico and Khachatourians, 2008). Extracellular fungal proteinases degrade the insect cuticle, which is composed of chitin and proteins, facilitating hyphae penetration into the host hemolymphoid. The key hydroxylating enzymes (Huarte-Bonnet et al., 2018b) also quickly assimilate hydrocarbons and lipid cuticular layers (Huarte-Bonnet et al., 2018a). This infiltration process involves cell walls, surface carbohydrates, and cell epitope(s) (Wanchoo et al., 2009; Zhang et al., 2011). Fungal or conidia are the main pathogenic factors of insect infection that get non-specifically absorbed through the insect epidermis. Under appropriate conditions, the conidia germinate to form hyphae and then secrete various insecticidal toxins (Lewis et al., 2009; Wanchoo et al., 2009). The *B. bassiana* toxins are primarily the secondary metabolites and small molecular compounds, such as beauvericin, bassianin, bassianolide, beauverolides, tenellin, oosporein, oxalic acid, calcium oxalate crystals, and many beauvericin analogs. Among these, mycelia-secreted beauvericin is one of the most important toxins (Molnár et al., 2010; Rohlfs and Churchill, 2011; Safavi, 2013). In addition, it possesses nematicidal activity (Xu et al., 2007). Interestingly, novel derivatives of beauvericin exhibit both cytotoxicity and insecticidal activity (Xu et al., 2007). Oosporein, apart from bactericidal and fungicidal activities, also inhibits tumor cell proliferation (Feng et al., 2015). It has been extensively studied for its wide spectrum of insecticidal properties and strong economic and environmental benefits (Figure 1).

The mechanism of pathogenicity varies with the type of toxin and host. The same toxin may have a different mechanism of pathogenicity and toxicity scale in different hosts. Thus, it is impossible to generalize the mechanism of action (Wang B. et al., 2012). However, the common consensus is that the fungal insecticidal effect is a cumulative result of several *B. bassiana* toxins. The insecticidal mechanisms involve several strategies, including the proliferation of virulence factors for continuous virulence, impeding the activation of the host immune system, disrupting the nerve conduction pathways, damaging the epidermis of the insect host to facilitate hyphae penetration, clogging of the spiracles the insect host, absorption of water and nutrients from the host body, and so on (Xiao et al., 2012; Ortiz-Urquiza and Keyhani, 2013; Pedrini et al., 2013). All these processes require high energy, which is met by the host insect hydrocarbons as a nutrient for the fungi (Kim et al., 2013; Huarte-Bonnet et al., 2015; Luo et al., 2015). Toxins also induce a range of symptoms in the host insect, including severe dehydration, abnormal behavior, lack of coordination, convulsions, hindered feeding, and metabolic disorders that eventually cause insect death (Chu et al., 2017).

Under appropriate conditions, the mycelia of the dead host produce numerous conidia (Feng et al., 2015). The epidermis of the parasite has already been devastated; the conidia are carried away with the air to infect other hosts, and a chain of infection continues (Pedrini, 2018). The spread of infection certainly requires the participation of other vectors, such as the leaves, roots, soil, and water, that are usually in close contact with insects. While these vectors create opportunities for rapid and widespread dissemination, they may also lead to non-targeted infection, including harm to beneficial insects.

As shown in Figure 1, most pathogenic fungi infect insects through the epidermis and then multiply in the hemolymph system. This is different from bacteria, viruses, and most other parasites which only trigger an infection after being ingested by the host (Ortiz-Urquiza and Keyhani, 2013). This unique mechanism of infection enables fungi to respond to various adverse environments, such as osmosis, hydrophobic barriers, electrostatic forces, relative humidity differences, and other biochemical factors similar to natural barriers, including phenols, esters, enzyme inhibitors, and proteins (Mazza et al., 2011; Gołębiowski et al., 2014). The figure shows that the fungal infection cycle not only depends on the successful penetration of the epidermis but also requires a dimorphic transition *in vivo*, i.e., the transformation of conidia into hyphae (Wang et al., 2017). The process can often be also manipulated at the genetic level by unannotated signaling pathways or downstream effector genes (Chu et al., 2017), which will be elaborated in the following text.

## Fungal Toxins Versus the Insect Immune System

The pathogenic process of *B. bassiana* in insect hosts is that of a facultative pathogen. Thus, infection is not a necessary physiological process of the fungal life cycle. The course of infection depends on the physiological state of the host, such as age, nutritional status, and several other physical and chemical factors, including ultraviolet light intensity, temperature, and humidity (Rangel et al., 2015a,b). A fungal infection normally takes about 6–14 days to kill the host insect (Charnley, 2003). Some insects with developed immune systems counter fungal infections by upregulating antifungal compounds and/or activating an innate immune response, including large amounts of reactive oxygen species (ROS), humoral melanization, and phagocytosis (Zibaee and Malagoli, 2014). For a successful infection, *B. bassiana* needs to resist the adverse physical and chemical environment and the host immune barrier (Fernandes et al., 2010; Shi et al., 2013; Barreto et al., 2016). These immune responses are often regarded as entry points or targets, which can widely affect the adaptability and the accessibility to the virulent fungi. Intricate immune responses of insect hosts involve many regulatory factors, which can be systematically explored to find specific targets.

Fungal toxicity involves direct and indirect factors (Figure 1). One such most-studied direct factor is melanin, which is a phenolic and/or indole compound with lipid or protein components. Melanin can counter UV, metal cytotoxicity, and lysozymes in the insect epidermis (Wilson et al., 2010; Shang et al., 2012). In general, melanin-producing fungi exhibit greater virulence than their albino mutants. Melanin also participates in the formation of antifungal compounds, such as defense peptides that counter the host response, and therefore it is classified as a direct factor (Langfelder et al., 2003). In addition, fungi upregulate the expression and the translation of oxidative stress response genes to counter the host immune response—for example, upregulation of superoxide dismutase accelerates the conversion of superoxide ions into molecular oxygen and hydrogen peroxide, thereby enhancing the *B. bassiana* tolerance to oxidative stress by eliminating the insect-produced ROS (Xie et al., 2010). The overexpression of heat shock factor 25 likewise facilitates fungus penetration and resistance against host immune attack even at 35°C in insect epidermis (Liao et al., 2014). *B. bassiana* also exerts other factors that can directly inhibit the host immune system. These are primarily the active proteins or secondary metabolites with insecticidal properties, such as cyclooligomer non-ribosomal peptides (beauvericin), cyclic peptides (beauverolides), and 2-pyridone tenellin (Molnár et al., 2010; Zhang et al., 2020a,b). These factors may also function as immunosuppressive compounds. Notably, a study in *Triatoma infestans* showed that, compared with the third day of infection, many of the antimicrobial peptide and defensin genes were significantly inhibited on the ninth day of infection (Mannino et al., 2019). Although the regulatory mechanism was not confirmed, it was largely due to fungal toxins (Baldiviezo et al., 2020). These findings suggest that fungi which secreted secondary metabolites can resist the immune system of the hosts at both genetic and non-physiological levels, which sets up the basis for toxin(s) pathogenicity.

The fungal intervention of the host insect immune system has also been linked to many indirect factors. Recently, oosporein, which is considered an indirect factor released by fungi, was shown to block the insect immune system. Oosporein involves three mechanistic pathways: (1) It inhibits the splitting of prophenoloxidase into polyphenol oxidase (PPO), which then hinders the activation of prophenoloxidase (PO); (2) It directly inhibits the expression of antifungal peptide gallerimycin (from *Gal* gene) at the post-transcriptional level; and (3) It blocks the antimicrobial peptide cascade response (Feng et al., 2015). Clearly, all these functions ultimately suppress the insect immune system. Researchers demonstrated that oosporein alone causes ∼20% mortality in the sap-sucking whiteflies, whereas oosporein combined with fungal conidia is more lethal, causing 92% mortality (Mc Namara et al., 2019). This suggests that oosporein promotes infection by inhibiting the immune and/or other defense mechanisms rather than *via* a direct cidal effect (Amin et al., 2011). Meanwhile, an RNA-Seq study, including Kyoto Encyclopedia of Genes and Genomes pathway annotation analysis, revealed that, at 24 h after infection, both the antioxidant (10) and peroxidase (7) genes were highly upregulated, indicating the importance of oxidative stress suppression during *B. bassiana* infection (Chu et al., 2016). Moreover, *B. bassiana* can inhibit the secretion of various antimicrobial compounds in insect cuticles, including quinones, such as methyl-1,4-benzoquinone, ethyl-1,4-benzoquinone, and 1-pentadecene (Villaverde et al., 2007; Yezerski et al., 2007; Joop et al., 2014). These compounds are natural immune barriers of insect hosts that must be overcome by *B. bassiana* for a successful infection. A protein named MCL1 also masks cell surface compounds, which hinders detection of hyphal bodies from insect hemocytes (Wang and St Leger, 2006). This process indirectly counters the insect immune system. Recent studies reported that there are multiple homologs of MCL1 in *B. bassiana* (Wang and St Leger, 2006). In a silkworm study, Nakahara et al. (2009) showed that entomopathogenic fungi infection somewhat affected the expression of hemocyte (granulocytes and plasma cells) immune genes (Nakahara et al., 2009). Another report showed that, compared with the control group, the total hemocyte number of *Galleria mellonella* was significantly decreased after infection (Bandani, 2004). Overall, these studies suggest that the immune system is one of the main targets of *B. bassiana* toxins. The host insect immunity played a potent selective force in fungi virulence evolution, most likely by upregulating the antioxidative stress genes that impede the activation of key immune pathways (Pedrini et al., 2015; Rafaluk et al., 2017).

## Genetic and Molecular Mechanisms Underlying Fungal Virulence

*Beauveria bassiana* benefits from its high genetic diversity. Recent studies showed that several genes/molecules can alter the virulence of *B. bassiana* depending on host and infection stage (Fang et al., 2004; Xiao et al., 2012). These genes/molecules can be potential targets to promote the application of *B. bassiana* as an effective and sustainable biological control agent. Table 1 summarizes the key genes and their corresponding mechanisms that play an important role in the fungal infection cycle. The list can be used as a comprehensive reference for selecting target genes and improve our understanding of virulence gene pleiotropy.

**TABLE 1:** Underlying genetic and molecular mechanisms of fungal virulence.

**Gene**	**Encoded protein**	**Function**	**Knock-out mutant phenotype**	**The process of participation**	**References**
*hyd1*	Hydrophobic protein	Modulate surface hydrophobicity, adhesion, and virulence	Inhibits virulence and conidia hydrophobicity	Adhesion, pathopoiesis, and cuticle degradation	Zhang et al., 2011
*hyd2*	Hydrophobic protein	Modulate surface hydrophobicity, adhesion, and virulence	Decreases conidia hydrophobicity and surface adhesion	Adhesion, pathopoiesis, and cuticle degradation	Zhang et al., 2011
*VLP4*	Vacuole-localized protein 4	Promote the melanization and the expression of *Pr1*	Increase virulence	Aerial conidia production and development, pathopoiesis	Chu et al., 2017
*Mdj1*	Heat shock protein	Manipulates several toxins at the transcriptional and/or post-transcriptional level	Mdj1 mutants have serious defects, such as damaged cell wall integrity, vulnerability to metal ions, and some physical and chemical pressures	Regulation of toxicity, conidia production, and transition from conidia to mycelia	Wang et al., 2017
*pks15*	Multifunctional enzymes	Synthesizes polyketides in fungi	Exhibits slow growth and decreases virulence	Overcome host immune responses, pathopoiesis	Toopaang et al., 2017
*PgpdA*	Promoter; transcription regulators	Encodes the promoter of glyceraldehyde-3-phosphate dehydrogenase of *Aspergillus nidulans*	Affects virulence	conidia production, and regulation of toxicity	Ruiz-Díez, 2002
*Ras1*, *Ras2*	Conserved hypothetical protein	Encode conserved hypothetical protein Ras	Affect virulence	Signal transduction and secrete toxins	Luo et al., 2014
*BbcreA*	Transcription regulators	Homologous genes of transcription regulators	Affects the virulence and the homeostasis of *B. bassiana*	Secrete toxins, hyphal extrusion, and conidiation	Luo et al., 2014
*Bbslt2*, *Bbhog1*, *Bbmpk1*	a-Glucose transporter; mitogen-activated protein	Encode an Slt2 family MAPK	Maintain the conidiation, cell wall integrity, and virulence	Secrete toxins, hyphal extrusion, and conidiation	Zhang et al., 2009
*trx1-6*	Thioredoxin antioxidants	Encode antioxidant activity thioredoxins	Reduced virulence, germination, conidiation, and stress tolerance	Host adhesion and conidia production	Zhang et al., 2015

Owing to the complex physiology and infection cycle, many genes of *B. bassiana* simultaneously participate in the infection cycle and basic processes such as conidia formation (Zhang et al., 2020a). Therefore, a gene often has multiple functions and virulence phenotypes. In gene mutation studies, the subsequent functional verification and examining the possible influence of such a candidate gene on the infection of *B. bassiana* can be intricate.

Most of such genes encode some virulence-related proteins or multipotent enzymes, which participate in all stages of *B. bassiana* infection, affecting host adherence, germination and penetration of conidia, cuticle degradation, colonization, and host death (Raya-Díaz et al., 2017). Therefore, examining the effects of gene deletion on virulence-related defects has become a prime theme of current research—for instance, *B. bassiana* has four genes encoding fungal pathogenicity, determining proteins with eight cysteine-containing extracellular membrane domains (Xiao et al., 2012). The transcription of these genes is partly controlled by bZIP-or C2H2-type transcription factors (TFs; Huang et al., 2015). Meanwhile, a BLAST search of insect pathogen genome against the *B. bassiana*–host interaction gene database (a collection of experimentally verified pathogenic, virulent, and effector genes from fungi and bacteria) revealed several G protein-coupled receptors, protein kinases, and TFs that are similar to entomopathogen genes (Gao et al., 2011). Notably, several of these genes, which are essential for *B. bassiana* infection, have strain-specific functions that vary with different hosts. In a review, Herrero has described these stage-specific genes in greater detail, especially their expression at various stages of *B. bassiana* infection (Herrero et al., 2012). Interested readers on the subject are welcome to consult Herrero et al. (2012) for further discussion.

Nutrient absorption and utilization are also important for *B. bassiana* parasitism involving genes such as forkhead transcription factor (*Fkh2*) (Wang et al., 2015), *Bbsnf1* (Wang et al., 2014), *Bbagt1* (a-glucoside transporter gene) (Wang J. et al., 2013), *Bbmpd*, *Bbmtd* (Wang Z.L. et al., 2012), and *BbCreA* (Luo et al., 2014). Although these genes do not directly regulate fungal virulence, they play an inseparable role in the infection process. A study showed that nearly 4,000 genes were differentially expressed in *B. bassiana* after 24, 36, and/or 48 h of infection (Chu et al., 2016). Importantly, nearly half of the upregulated genes were of putative secretory proteins (PSPs) that affect fungal virulence (Stergiopoulos and de Wit, 2009; Collette and Lorenz, 2011)—for instance, putative methyltransferase BbmtrA affects conidial viability, fungal growth, and virulence (Qin et al., 2014). Evidently, at least some of the putative secretory proteins are of particular importance, and therefore proteins of unknown function, such as VLP4 and many more small PSPs, need proper investigations for their role in fungal virulence.

In addition, some powerful gene promoters can stably enhance the expression of host target genes increasing the insecticidal virulence of *B. bassiana* (Ruiz-Díez, 2002)—for instance, in filamentous fungi *Aspergillus nidulans*, *PgadA* is expressed under the promoter of glyceraldehyde-3-phosphate dehydrogenase (Liao et al., 2008). Similarly, some genes encoding for the conserved hypothetical protein Ras, such as *Ras1* and *Ras2*, showed different insecticidal virulence to the larvae of *G. mellonella* in different strains of *B. bassiana* (Luo et al., 2014). Certain homologous genes of transcription regulators, such as the carbon catabolite repressor transcription factor homolog (BbcreA), can also affect the virulence and homeostasis of *B. bassiana* (Luo et al., 2014).

Like other pathogenic fungi, *B. bassiana* has several GATA-type TFs. Apart from virulence gene regulation, these are also involved in multiple functions, including nutrient uptake, mating-type switching, and chromatin rearrangement (Xiao et al., 2012). In *B. bassiana*, bZIP- and C2H2-type TFs that show a higher activity under alkaline conditions behave similar to GATA-type TFs and regulate pathogenicity (Huang et al., 2015). Therefore, it seems that some fungal virulence-related genes are majorly governed by TFs, and identifying many more regulatory components can establish good biochemical and molecular data that can further reveal the pathogenicity of *B. bassiana*.

## Genetic Engineering Strategies to Improve Fungal Toxicity

In addition to directly involved genes, some genes indirectly participate in toxicity. These genes can adjust the host–fungi interaction in favor of the pathogen, which increases the fungal virulence (Rohlfs and Churchill, 2011; Mascarin and Jaronski, 2016). Genetic engineers believe that such genes can be the prime targets to increase virulence. We have listed several such genes in Table 2 as a reference for such efforts.

**TABLE 2:** Target genes that improve virulence by genetic engineering strategies.

**Modified gene**	**Mode of action**	**Effect**	**References**
Pr1A	Fused CDEP1 with Bbchit1 and Pr1A	LT50 decreased by 25% LC50 decreased by 25%	Fang et al., 2009; Fan et al., 2010
Cyt2Ba	Transformed pBARGPE1-Cyt2Ba into blastospores	Infection rate increased LT50 decreased Reproductive rate decreased	Deng et al., 2019b
TMOF	Introduced Aea-TMOF into *B. bassiana*	LT50 decreased by 15% LC50 decreased by 40%	Kamareddine et al., 2013
TMOF	Fused CP of TMV with TMOF	LC50 decreased	Borovsky et al., 2006
Spn43Ac	Introduced Spn43Ac of *Drosophila* into *B. bassiana*	LT50 decreased by ∼24% LC50 decreased by 300%	Yang et al., 2014

With the advancement of biotechnology, genetic engineering strategies seem to be the most convenient method to improve fungal virulence. However, there are conspicuous controversies. Many believe that genetically modified toxins are not safe and therefore lack wide acceptance; the products of genetic modification may also not be stable, causing uncontrollable consequences due to unknown events (Mascarin and Jaronski, 2016). Therefore, genetic modification studies usually need a long time to apply in the field, and the commercialization takes even longer. On the contrary, synthesizing new chemical insecticides requires an investment of at least 250 million dollars and several years, which can be a huge economic burden in many countries (Glare et al., 2012). Genetic strategy can improve the adverse characteristics of *B. bassiana* such as low toxicity, slow effect, and its adaptability to harsh environments, including heavy rain and ultraviolet (St Leger and Wang, 2010). Recent reports showed that genetic strategies that improved fungal toxicity are safe; however, safety must remain a priority in the future, too.

To find differential genes that may increase virulence requires large-scale transcriptome sequencing and screening (Glare et al., 2020). This strategy has become a common theme of the fungal virulence field. Compared to other insect pathogens, *B. bassiana* has great genetic diversity, showing a significant diversity among isolates (Lee et al., 2018). Therefore, finding appropriate target genes could be a challenging task. However, past efforts have already shown that some of the protease-related genes and their transcription regulators may greatly improve the virulence of *B. bassiana*. Presently, differential genes are scanned at three levels: (1) screening the differentially expressed genes (DEGs) of *B. bassiana* before and after pathopoiesis (Shi et al., 2013), (2) screening the DEGs in different natural strains of *B. bassiana* with a different pathogenicity (Luo et al., 2015), and (3) forcing mutation in *B. bassiana* by exposing the mycelia to abiotic stresses (such as strong acid, strong alkali, strong ultraviolet, hypoxia, and nutrient deficiency) to screen for DEGs that affect virulence (Rangel et al., 2015b). These themes can help researchers to quickly screen out suitable mutant strains and mutant genes. Regardless of the screening method, the wide natural genetic variation within *B. bassiana* provides a huge possibility of DEG screening (Pedrini, 2018) that can also be used for subsequent genetic engineering strategies.

The other common theme is to improve virulence through direct gene manipulation or gene recombination of protoplast fusion (Raya-Díaz et al., 2017). CRISPR-Cas9 technology-based novel RNA-guided mutagenesis and genomic data mining can establish good recombinant DNA techniques for genetic engineering strategies (Xiao et al., 2012). Meanwhile, protein engineering, involving direct evolution, sequential error-prone PCR, DNA shuffling, and so on, is another viable method to tailor the toxicity-related proteins or enzymes of *B. bassiana*. In brief, heterologous transgenic expression and/or fusion protein technique(s) have been successfully applied to improve fungal toxicity. Cytolytic δ-endotoxin (Deng et al., 2019b) and trypsin-modulating oostatic factors (TMOFs; Ortiz-Urquiza and Keyhani, 2015) are examples of heterologous transformation that improved the toxicity of insecticidal toxin genes, albeit with a suitable promoter and an expression vector. TMOF-CP, a fusion of TMOF with the tobacco mosaic virus coat protein (CP), is an example of a fusion protein approach. This transgenic CP showed little effect on the growth of plant leaves, while it produced large amounts of RNA and protein in infected plant cells (Borovsky and Meola, 2004). The improved production efficiency of CP-TMOF chimeras also showed better larval lethality (87.5% at 26.4 μM, equivalent to 140 ng/ul TMOF or 2.33 μg/μl CP-TMOF), exhibiting a 7.5-fold improvement over TMOF alone (Borovsky et al., 2006).

Though the genetic strategies have increased fungi virulence, mass production remains a problem. Nearly 90% of commercially available fungal insecticides are produced by convenient asexual propagation methods using liquid media, which requires less culture time (Mascarin and Jaronski, 2016). However, in this technique, only about 10% of the offspring exhibit genetically modified phenotypes, which is not conducive enough (Mascarin and Jaronski, 2016). A market research report found that price is also a major factor; the price of genetically modified biocides is much higher than that of field strains, hampering consumer acceptance (Mckinnon et al., 2017).

## Newly Discovered Toxins and Enzymes

In recent years, many novel *B. bassiana* toxins or enzymes have been discovered, which are now being industrialized to meet the market demand. Most secondary metabolites/toxins possess different industrial significance according to their potential in various agricultural and pharmaceutical applications (Pedrini et al., 2015). The growing understanding of the evolution of toxin diversity will ease the further development of *B. bassiana* bioinsecticide in a more eco-friendly and efficient way.

Khan et al. extracted Bb70P, a toxic protein to insects, from the *B. bassiana* 70 strain. Bb70P is approximately 35.5 kDa, with an isoelectric point of 4.4 (Khan et al., 2016). Purified Bb70p is most active in weakly acidic media and remains active up to pH 4–10 (Khan et al., 2016). Hemocoel injection bioassays showed that the LC_50_ of Bb70p against *G. mellonella* was 334.44 μg/larvae (Kavanagh and Reeves, 2004). It was suggested that, after treatment, Bb70p triggers the melanism of *G. mellonella*, changing the body color of the insect from black or brown, as an immunoreaction of the host. Moreover, Bb70p transformed inactive PO into active PPO (Kavanagh and Reeves, 2004), suggesting the importance of host insect immune invasion for fungal pathogenesis.

Lysyl-tRNA synthetases (Krs), a family of aminoacyl-tRNA synthetases, have a small N-terminal tRNA anticodon binding domain, a large C-terminal catalytic domain, and three conserved sequence motifs (Desogus et al., 2000; Guo and Yang, 2014; Oka et al., 2015). The cytoplasmic Krs of *B. bassiana* support conidia germination and dimorphic transition. As an independent virulence factor, the deletion of Krs significantly decreased the aerial conidiation (∼47%) and conidia tolerance to wet–heat stress (∼15%) and ultraviolet (∼46%) (Zhu et al., 2017). Importantly, the virulence of the Krs-deleted strain against *G. mellonella* was 60% lower than the wild-type strain. This was attributed to delayed conidial germination and reduced extracellular Pr1 enzyme activity, which reduced mycelial penetration into the host epidermis (Zhu et al., 2017).

Vegetative insecticidal proteins (Vips), with intense insecticidal activity, were originally isolated from *B. thuringiensis* (Schnepf et al., 1998). Vip3A showed a specific insecticidal activity in Lepidopteran insects (Mesrati et al., 2005; Milne et al., 2008). Qin et al. (2010) used the Vip-encoding gene to construct Vip3Aa1-transformed *B. bassiana* BbV28 and induced Vip3Aa1 expression in both mycelia and conidia. The study found that conidia expressing Vip3Aa1 were the main source of insecticidal virulence, at least during the first 3 days of infection (Qin et al., 2010). Compared with the wild-type strain, the Vip3A1-transformed strain not only exhibited improved insecticidal spectrum and virulence to pests but also infected the host by surface infiltration and *per os* (Qin et al., 2010). Besides this, the Vip3Aa1 expression under the promoter (*Phyd1*) of the *B. bassiana* class I hydrophobin gene (*hyd1*) increased the insecticidal virulence by 9.8-fold (Wang Z.L. et al., 2013).

There is a readily available toxin, a chitosanase-like protein (Bclp), which was isolated from the *B. bassiana* 618 strain. This 28-kDa toxin protein is highly hydrophilic, with an isoelectric point of 4. It kills *G. mellonella via* melanization (Fuguet et al., 2004). It also primarily damages the epithelial cells of the epidermis and the trachea. The damaged outer epidermis leads to hemocyte infiltration and injury (Fuguet and Vey, 2004). Based on these features, this protein is considered a potent toxin; however, it is vulnerable to high temperatures (60 and 115°C) and proteases (Fuguet et al., 2004).

Other studies tested genetically fused toxins to generate higher virulence—for example, the subtilisin-like serine protease gene, *CDEP2*, contains an open reading frame of 1,137 bp and can be translated into a 379-amino-acid protein (3.9 kDa) with an isoelectronic point of 8.21 (Xia et al., 2009). Xia et al. (2009) fused this gene with the *B. thuringiensis cry1Ac* gene into plasmid pHT315 to form a plasmid pHAc-CDEP2, under the promoter *cry1Ac*. After electroporation into *B. thuringiensis*, the recombinant gene was translated into a 130-kDa cry1Ac protein and a 76-kDa CDEP2 protein, which significantly improved the fungal toxicity against the third instar larvae of *Helicoverpa armigera* (Hübner). In another research, a scorpion neurotoxin peptide, *aaIT*, and the *Pr1A* were fused for a simultaneous and stable expression in *B. bassiana* strain 13 to improve the lethality of the original toxins. *B. bassiana* that expressed *aaIT* alone and those co-transformed with *aaIT* and *Pr1A* showed an LT_50_ of 4.5 days and 4.75 days, respectively, against *Dendrolimus punctatus* Walker. Compared to the wild-type strain (7.5 days), this was 40 and 36.7% reduction, respectively. Similarly, the LT_50_ against *G. mellonella* was 3.25 and 3.4 days, a decrease of 24.4 and 20.9%, respectively, compared to the wild type (4.3 days) (Lu et al., 2008; Deng et al., 2019a). These results suggest that the interaction between the protein products should be seriously considered before generating the corresponding fusing genes which may be vulnerable to insect proteases.

The high genetic diversity of *B. bassiana* suggests that it is not a monophyletic strain, and therefore further genome-wide phylogeny of *B. bassiana* is necessary (Rehner et al., 2006; Ghikas et al., 2010). Herein we list the characteristics of genomes from four different strains in Table 3, which allows the comparison and discovery of new toxins. Newly discovered toxic proteins, which may function like chemical pesticides, can improve the entomopathogenic activity of *B. bassiana*. Further *in vivo* and *in vitro* studies could help drive the commercialization of these novel toxins.

**TABLE 3:** Characteristics of genomes of four different *Beauveria bassiana* strains.

**Species**	**Strain**	**Accession number**	**Genome length**	**Number of introns**	**Genome size without introns**
*Beauveria bassiana*	e17	KT201149	29,944	3	25,859
	Bb13	EU371503	29,961	3	25,807
	Bb147	EU100742	32,263	5	25,740
	k4	KT201148	28,816	2	25,712

## Conclusions and Future Perspectives

In pest control, the *B. bassiana* biological control agent has the unique advantage of a broad-spectrum insecticidal activity against most agricultural pests and medically important arthropods (Jackson and Jaronski, 2009; Montalva et al., 2016). Although *B. bassiana* has been a proven effective biopesticide for decades, there is still some resistance to its commercial application: (1) Increasing taxonomic complexity within the genus *Beauveria* has made the true taxonomic status of many commercial and experimental *Beauveria* strains uncertain; (2) A wide range of phenotypic characteristics have not yet been reported; and (3) Insects can develop resistance to *B. bassiana* by upregulating the expression of certain genes, such as P450s and epidermal protein genes. *B. bassiana* has evolved a series of effective virulence mechanisms involving the breach of the epidermal barrier and host insect immune attack systems, such as secretory epidermis-degradative enzymes, mechanical penetration, avoidance of being engulfed by phagocytes, and inhibition of the release of active immune substances (Kirkland et al., 2005; Almudena et al., 2013; Pedrini et al., 2013). However, a growing number of studies found many adverse factors that may limit the use of *B. bassiana* as a biopesticide, including a relatively long time to kill the target insect, effect on non-target invertebrates, allergies in humans, dependence on ambient temperature and humidity, growing insect resistance, and uncertain taxonomic status of the fungi. Fascinatingly, genetic engineering can specifically improve these deficiencies; however, most of these strategies have so far been restricted to the laboratory and need to be extensively popularized and applied.

The discovery of new biocides or toxins is largely serendipitous or coincidental, but for now, improving the toxicity of fungus based on genetic engineering strategies may be the most appropriate option. Fungal secondary metabolites are also of additional concern. They not only affect the insecticidal process but also intervene with the host plant pathophysiology, as shown in numerous crop species (Mckinnon et al., 2017). In addition, some fungal secondary metabolites have potential medical applications (Glare et al., 2020).

Since the vertebrate and the invertebrate immune pathways are not the same, manipulating the insect immune system for fungal invasion and infection may be a desirable method. A natural insecticide such as *B. bassiana* may ensure human safety and provide an environment-friendly pest control strategy (Fernandes et al., 2010; Barreto et al., 2016). Besides this, along with existing chemical insecticides or synergists, novel researched toxins, after a thorough understanding of their structural properties, *in vitro* insecticidal mechanism, insecticidal efficacy, effects on other non-target organisms, and increasing toxin production by *in vitro* synthesis, may be an effective way to ameliorate the development of insecticide resistance and improve insecticidal efficiency.

## Author Contributions

HW and HP participated in writing–review and editing. WL and PC participated in visualization. MG participated in supervision and project administration. MG and PC participated in funding acquisition. All the authors have read and agreed to the published version and approved the final manuscript.

## Conflict of Interest

The authors declare that the research was conducted in the absence of any commercial or financial relationships that could be construed as a potential conflict of interest.

## Publisher’s Note

All claims expressed in this article are solely those of the authors and do not necessarily represent those of their affiliated organizations, or those of the publisher, the editors and the reviewers. Any product that may be evaluated in this article, or claim that may be made by its manufacturer, is not guaranteed or endorsed by the publisher.
